# Translation and validation of menopause quick 6 (MQ6) into the Malay language

**DOI:** 10.1186/s12875-024-02342-3

**Published:** 2024-03-22

**Authors:** Anusha Manoharan, Megat Muhammad Harris, Beh Hooi Chin, Koh Wen Ming, Zamzurina Asmuee, Norafini Salamon, Jerampang Pefer, H. Radhiyah, M. Nadia Hamimmah, Susan Goldstein, Shamala Ramasamy, Chandrashekhar T. Sreeramareddy

**Affiliations:** 1 Klinik Kesihatan Bandar Botanic, Bandar Botanic, Selangor Malaysia; 2 Klinik Kesihatan Jinjang, Kuala Lumpur, Malaysia; 3 The Department of Primary Care, University Malaya, Kuala Lumpur, Malaysia; 4 Klinik Kesihatan Rawang, Rawang, Malaysia; 5 Klinik Kesihatan Kuang, Kuang, Malaysia; 6 Klinik Kesihatan Jalan Merbau, Sarawak, Malaysia; 7 Klinik Kesihatan Tanah Puteh, Sarawak, Malaysia; 8 Klinik Kesihatan Tanglin, Kuala Lumpur, Malaysia; 9 Klinik Kesihatan Putatan, Sabah, Malaysia; 10 Department of Family and Community Medicine, University of Toronto, Toronto, Canada; 11 Department of Psychology, International Medical University (IMU), Kuala Lumpur, Malaysia; 12 Department of Community Medicine and Public Health, International Medical University (IMU), Kuala Lumpur, Malaysia; 13 Centre for Translational Research, Institute for Research Development and Innovation, International Medical University, Kuala Lumpur, Malaysia

**Keywords:** Menopause, Questionnaire, Primary care, Treatment, Validation study

## Abstract

**Background:**

Inquiring conservative Asian women about their menopausal symptoms is often challenging in crowded primary healthcare clinics. Furthermore, the subject matter is culturally sensitive to most Malaysian women. Hence, the translation of the MQ6 into Malay is crucial to enable self-administration, eliminating the necessity for interviewers and mitigating potential respondent shyness.

**Methods:**

The Menopause Quick 6 (MQ6) questionnaire was translated into the Malay language with an addition of an item, henceforth termed MQ6 (M). Forward and backward translation was performed. Face and content validity were conducted. MQ6 (M) was self-administered to 400 women aged between 40 and 60 attending six primary healthcare clinics in Malaysia. To ascertain the reliability for MQ6 (M), corrected Item-Total Correlation, Squared Multiple Correlation, Cronbach’s Alpha if the Item is Deleted, and Kuder-Richardson Reliability Coefficients (KR20). Exploratory factor analysis was done to determine its’ construct validity.

**Results:**

The outcome of the validation was satisfactory. By the Lawshe method, the content validity ratios ranged from 0.6 to 1.0 and the content validity index was 0.914. The Internal consistency for the MQ6(M) Cronbach’s alpha was 0.711 while Kuder-Richardson Reliability Coefficients KR20 was 0.676. Factor loading of all four items is above 0.70, indicating a well-defined structure. Whereas factor loading for three items fell within the range of 0.50–0.69 indicating a practically significant threshold for a new questionnaire.

**Conclusion:**

The MQ6 (M) has acceptable reliability and construct validity to be considered as a self-administered screening tool in primary care clinics in Malaysia.

**Supplementary Information:**

The online version contains supplementary material available at 10.1186/s12875-024-02342-3.

## Background

Menopause occurs due to declining hormones with increasing age when women start experiencing a range of symptoms [1]. However, symptoms and their severity vary by geographic region due to the perceptions of women and psychosocial factors [2]. Women in midlife and those living into older age are affected by menopause which may impact women’s quality of life [3]. Therefore, healthcare providers are required to appropriately advise women by suitably assessing their menopausal symptoms [4]. Menopausal hormone therapy has been proposed to be effective management of vasomotor symptoms, genitourinary, syndrome of menopause, as well as bone protection, a quick assessment tool would be of utility to make a patient-centered assessment to provide evidence-based treatment choices [5, 6].

Primary care physicians are the first point of care for all age groups and provide services such as chronic disease management [7]. Women usually receive advice or treatment for menopause symptoms from a primary care physician [8, 9]. Thus, it is imperative to have a valid screening tool in primary care clinic settings to assess menopausal symptoms to offer treatment. Existing validated menopausal questionnaires in English such as the Menopause Rating Scale (MRS) [10] and Menopause-Specific Quality of Life Questionnaire (MENQOL) [11] are widely used in specialized obstetrics and gynecology clinics or research settings. The MRS and MENQOL can perhaps be used in primary care settings as well. However, instruments that are relatively shorter, and take less time are suitable for a quick assessment of menopausal symptoms. Such instruments are beneficial for busy primary care clinics that are attended by midlife women for various ailments including chronic disease care. In addition to quick assessment such tools are proposed to be useful to make treatment decisions on hormonal or non-hormonal treatments [12].

The Menopause Quick 6 (MQ6) has been proposed as an alternative screening tool for brief and fast self-administered screening of women attending primary care clinics for chronic disease care [12]. Six items and responses in binary format as the presence or absence of its symptoms instead of rating their severity are beneficial for making a quick assessment. The prevalent menopausal symptoms among Malaysian women differ from the Western population. Hot flushes and night sweats are more frequently reported in Western women but musculoskeletal symptoms are commonly reported by Malaysian women [2]. Therefore the need to modify the MQ6 by adding a question about musculoskeletal symptoms that are more frequently reported in our community to assist treatment decisions [9, 13, 14]. This study aims to translate and validate the proposed menopausal assessment tool MQ6(M) to be used in primary health care clinics to identify common menopausal symptoms among middle-aged women.

## Methods

### Design, setting, and participants

A cross-sectional questionnaire survey was done among middle aged women attending the Ministry of Health, Malaysia’s primary care clinics (also known as Klinik Kesihatan). Participants were Malaysian women, with an age range from 40 to 60 years who were eligible to participate in the survey. However, women with history of heart ailments, psychiatric conditions (self-reported), a history of drug or alcohol abuse, cancer treatment, premature ovarian failure or genital malformation, and artificial menopause either medical or surgical, those on hormone replacement therapy, and pregnant, lactating women were the exclusion criteria.

### Sample size calculation

We calculated the sample size for the survey on menopausal symptoms among women attending health clinics. For 95% confidence limits (Z = 1.96), an allowable marginal error of 5%, and an anticipated proportion of 70% for the presence of menopausal symptoms. The minimum sample size was 322 using the formula, and after allowing for a 20% non-response rate, the final sample size was 386 using the formula. However, the sample required for the validation of the questionnaire for meaningful and interpretable values is 200 [15].

Mundfrom et al. recommended that the minimum sample size should be determined based on the various variables-to-factor ratios, taking into consideration both high and wide communality [16]. In addition, Kline (2023) suggested > 200 sample size is ideal for a validation study [17]. Therefore, for this validation study, with a 7-items single-factor questionnaire, the minimum sample size calculation is taken as 400 after allowing for a 20% non-response rate.

### Sampling method

Six health clinics i.e. two each from the states of Selangor, Sarawak, and Sabah were purposively chosen to provide representation of a multiethnic population consisting of ethnic Malays, Chinese, Indians, and indigenous groups. The states of Sabah and Sarawak and a part of Peninsular Malaysia have indigenous people which consist of ethnic minorities. In each clinic all successive women attending the outpatient clinics were invited to participate in the study. The number of women recruited for the survey ranged in each of the clinic ranged from 50 to 70.

### Menopause Quick 6 Malay, MQ6(M)

The questions from the Menopause Quick 6 (MQ6) were adapted from the proposed instrument available freely online at https://mq6.ca/mq6-assessment-tool/. MQ6 was proposed as a quick and efficient tool to check for the presence of menopausal symptoms and to assist the physicians to make treatment decisions based on an algorithm [12]. However, the original tool had not yet been validated. After obtaining written permission from the author and publisher, we translated and adapted the MQ6 to suit the local population’s sociocultural context and variability of menopausal symptoms in Malaysian population. The original MQ6 contains six questions consisting of the chief menopausal symptoms all under one domain. The scale of measurement is a binary response as ‘yes’ or ‘no’ for most items. Total score was not recommended since the tool is used to make treatment decisions based on an algorithm. A question about muscle and joint pain was included to reflect the Malaysian population [14] (supplement A).

### Translation of MQ6 English to the Malay language

We use forward and backward translation methods to translate the proposed MQ6 original (unvalidated) in English into the Malay language version MQ6(M) [18]. At first, a bilingual expert did the forward translation into the Malay language version. The Malay language version was further reviewed by a researcher who is proficient in the Malay language and is conversant with the Malaysian socio-cultural context. In the second step, a blind bilingual expert backtranslated the Malay language version into the English language. Following this, all translators created a final, consolidated version and approved the final version.

### Validation of MQ6(M)

The MQ6 (M) questionnaire went through a validation process for its relevance and clarity. Face validity was done by 20 participants including the women, doctors, and other healthcare staff at one of the survey sites. Participants’ comprehension of the items was assessed by the time to completed MQ6 (M). Any feedback and suggestions were also gathered. The content validation was done to improve the questionnaire’s adequacy, accuracy, and appropriateness. A panel of 10 experts, eight primary care physicians, and two obstetricians and gynecologists were invited to participate. A cover letter was sent electronically with clear instructions on how to rate each item. Amendments were made based on the feedback from expert panels. The content validity ratios (CVR) and content validity index (CVI) were calculated as per the Lawshe method [19]. The CVR ranged from 0.6 to 1.0 (0.6 for item 2) and CVI was 0.914. The CVI index of 0.914 is an acceptable value with at least 9 expert raters according to Lynn (1986) [20].

### Participants recruitment

Women attending the primary care clinics selected for this survey were assessed for eligibility based on the medical information that was available at the clinic and that provided by the women. Those women who were eligible were invited to participate in our study after a detailed explanation about the study purpose and questionnaire to be self-administered. Women who consented completed the survey questionnaire containing demographic information, medical history, and the MQ6(M). A subsample of 30 women who completed the questionnaire agreed and completed the same questionnaire after two weeks for test-retest reliability.

### Ethical approval

This study does not violate the policies and/or procedures established by the journal such as those described in ‘Specific Inappropriate Acts in Publication Process. Ethical approval was obtained from the Medical Research Ethics Committee of Malaysia to conduct this study. (NMRR ID-21-02265-2S0(IIR)) and conforms to the provisions of the Declaration of Helsinki. followed current regulations on the protection of personal data. This study also followed current regulations on the protection of personal data, in which participant information sheets assured anonymity and confidentiality. Informed consent from participants was obtained.

### Statistical analyses

Descriptive statistics of the study participants’ demographics and responses to the MQ6(M) questionnaire were calculated. The following reliability statistics were estimated: Corrected Item-Total Correlation, Squared Multiple Correlation, Cronbach’s Alpha if Item is Deleted, and Cronbach’s Alpha. In addition, we also estimated Kuder-Richardson 20 (KR20) for binary response options of MQ6(M). For construct validity, we have conducted a factor analysis of a matrix of tetrachoric correlation as it is more appropriate for binary responses. We estimated the kappa statistic between each item for test-retest reliability as the responses were binary. On StataMP11 we analyzed tetrachoric correlations by adjusting the correlation matrix to be a positive semidefinite. We did a factor analysis on the matrix using *‘factormat*’ and selected the iterated principal factors as the estimation method on Stata MP(version 11) [21].

## Results

Table 1 shows the demographics of women surveyed. Of the sample of 400 women, 235 (58.7%) were aged between 50 and 60 years (Mean age 52, SD 6.3 years), 73.4% were currently married, over a third were Malay women and about a third were indigenous communities of Sabah, and Sarawak of East Malaysia and 61.3% of them were Muslims.

**Table 1:** Demographic characteristics of survey participants

Independent variables	Frequency *n* (%) (*N* = 400)
**Age group**	
40–49 years	165 (41.3)
50–60 years	235 (58.7)
**Marital status**	
Currently Married	295 (73.8)
Never married	23 (5.8)
Divorced	25 (6.3)
Separated	6 (1.5)
Widowed	51 (12.6)
**Ethnicity**	
Malay	151 (37.8)
Chinese	61 (15.3)
Indian	45 (11.3)
Sabahan	90 (22.5)
Sarawakian	37 (9.3)
Others	16 (4.0)
**Religion**	
Islam	245 (61.3)
Budhha	38 (9.5)
Hindu	40 (10.0)
Others	71 (17.8)
Christian	6 (1.4)
**Highest education level**	
No education	24 (6.0)
Primary school	72 (18.0)
Secondary school	212 (53.0)
High educational	89 (22.3)
Others	3 (0.8)

The reliability statistics of each of the MQ6(M) items are shown in Table 2. The overall reliability of the scale by Cronbach’s alpha was 0.711 while KR20 was 0.676. Test-retest reliability was conducted within two-week interval. The established index was between 0.8 for one item and 1.0 for the other six items of the MQ6(M). Internal consistency, the value of Cronbach’s alpha for the 7-item questionnaire yielded 0.711. The reliability is acceptable taking into consideration the number of items and the new questionnaire [22]. Most of the inter-item correlations were moderately correlated (< 0.30) except a few are within acceptable range [23, 24]. Of all seven items (Item 5), only one item has a corrected item-total correlation value of less than 0.30, a recommended threshold [25]. Nevertheless, the highest Cronbach Alpha if item deleted value is 0.707 for item 5 which only contributes a variance of 0.147.

**Table 2:** Reliability statistics of MQ6(M) items

	Corrected Item-Total Correlation	Squared Multiple Correlation	Cronbach’s Alpha if Item Deleted	Cronbach’s alpha	Test-retest validity
Any changes in your periods?	0.379	0.175	0.689	0.6902	0.8
Are you having joint stiffness or muscle aches?	0.442	0.229	0.671	0.6721	1.0
Are you having any hot flashes?	0.559	0.351	0.637	0.6470	1.0
Any vaginal dryness or pain, or any sexual concerns?	0.426	0.218	0.676	0.6725	1.0
Any bladder issues or incontinence?	0.294	0.147	0.704	0.7070	1.0
How is your sleep?	0.352	0.152	0.693	0.6933	1.0
How is your mood?	0.504	0.281	0.655	0.6587	1.0
Overall test scale by Cronbach’s alpha				0.711	
Kuder-Richardson Reliability Coefficients KR20				0.676	

Exploratory Factor Analysis (EFA) of the seven items of the MQ6(M) is shown in Tables 3 and 4 shows factor loadings. A unidimensional factor was extracted based on the eigenvalue above 1.00. Only one factor displayed an above eigenvalue of 1.00, which was 3.240. The total variance explained extracted was 66.77% (Table 3). Kaiser-Meyer-Olkin (KMO) measure of sampling Adequacy was 0.78 was good and Bartlett’s test of Sphericity was χ^2^ = 428.4, *df* = 21, *p* < 0.001 which was below the threshold of 0.05 (reported if *N* > 200). Therefore, of all the 7 items analyzed, only 1 factor is being produced. Factor loadings of all 4 items are above 0.70, indicating a well-defined structure. Whereas factor loading for 3 items fell within the range of 0.50–0.69 indicating a practically significant threshold. Based on the results of the reliability assessments, it has been decided that no items should be discarded.

**Table 3:** Results of unrotated factor analyses using ‘factormat’ C analyses

Factor	Eigenvalue	difference	proportion	cumulative
Any changes in your periods?	3.24	2.87	1.00	1.00
Are you having joint stiffness or muscle aches?	0.37	0.22	0.12	1.12
Are you having any hot flashes?	0.16	0.03	0.05	1.16
Any vaginal dryness or pain, or any sexual concerns?	0.13	0.22	0.04	1.2
Any bladder issues or incontinence?	-0.10	0.11	-0.03	1.12
How is your sleep?	-0.20	0.16	-0.06	1.11
How is your mood?	-0.36	-	-0.11	1.00

chi2(21) = 1288.97 Prob > chi2 = 0.0000

**Table 4:** Factor loadings (pattern matrix) and unique variances

Factor	Factor 1	uniqueness
Any changes in your periods?	0.5764	0.6677
Are you having joint stiffness or muscle aches?	0.7029	0.5059
Are you having any hot flashes?	0.8078	0.3474
Any vaginal dryness or pain, or any sexual concerns?	0.7109	0.4947
Any bladder issues or incontinence?	0.5853	0.6575
How is your sleep?	0.5652	0.6805
How is your mood?	0.7707	0.4060

## Discussion

### Main findings

The survey of patients attending primary care clinics was used to validate the MQ6 originally proposed for use in primary care to assist with decisions on hormonal treatment for menopausal symptoms. The MQ6 in the original English language has not yet been validated, allowing us to translate and validate a modified version of the original MQ6 into a Malay language version i.e., MQ6(M). Our validation study demonstrated acceptable psychometric properties as a quick tool applicable in primary care settings. The MQ6(M) had high content validity and repeatability. Malaysian women did not express any concerns such as non-comprehensibility or ambiguity of items in MQ6(M) or acceptable reliability.

### Comparison of MQ6 (M) with MRS

The reliability of a proposed new instrument in the Malay language has an acceptable internal consistency as recommended though a higher value is desirable for an instrument to assess MQ6(M) intended for use in primary care settings [26, 27]. A comparable tool for menopausal symptom assessment MRS in Indonesian Bahasa [28] and Malay language [29] though had a higher reliability than a newly proposed MQ6(M). However. MRS international versions varied in Cronbach’s alpha (0.6–0.9) [30]. MRS had 11 items while MQ6(M) has 7 items of which all except item-7 about mood fall under somatovegetative and urogenital domains of MRS. The lower reliability of MQ6(M) is comparable to international language versions of MRS which were reported to have lower reliability for urogenital and somatic compared to the psychological domain [30]. However, MRS measured menopausal symptoms on a Likert scale to measure severity while MQ6(M) is purposed to make a quick assessment for making treatment decisions and does not cover all menopause symptoms and uses a binary scale of measurement. The reliability measures used were nearly the same and were an acceptable range.

A new tool proposed a well-defined structure and none of the proposed items were omitted after EFA suggesting that the new tool could be adapted for the Malaysian setting after further testing. The MRS comparable tool to MQ6(M) is known to have factor instability when tested in different languages in multi-country versions [30]. Such instabilities in factor structures are known to occur due to sociocultural differences that determine the perceptions of physiological changes that occur during menopause[ [29]. Due to the binary scale of measurement and a lack of domains of items we did not perform a confirmatory factor analysis to determine factor structure. Nevertheless, reported factors of instability and geographic variability of perception of menopausal symptoms should be kept in mind while interpreting MQ6(M). The new quick assessment tool be further tested on a population-based sample of mid-life women rather than those seeking healthcare at primary care clinics. The validity of the original source MQ6 in English should also be established to enable primary care researchers to translate and validate in international language versions.

### Limitations

We should interpret the results of the validation of the MQ6(M) considering some limitations. First, the hospital-based study is likely not a representative sample Malaysian population in terms of ethnic distribution. Self-administered questionnaires collecting information on menopausal symptoms are subject to recall bias. Misreporting of symptoms is also possible due to emotional status, living circumstances, misconceptions about sexuality, and perceptions of ‘hot flushes’ in the tropical weather prevailing in Malaysia.

In conclusion, the MQ6(M) tool, translated into Malay language, had an acceptable psychometric property such as reliability, content and construct validity. The MQ6(M) with the inclusion of additional item on musculoskeletal symptoms has a potential for application in primary care clinics as a quick assessment tool for menopausal symptoms among Malaysian women.

### Electronic supplementary material

Below is the link to the electronic supplementary material.

Supplementary Material 1

## Data Availability

The datasets used and/or analysed during the current study are available from the corresponding author on reasonable request.
