# Correction: Translation and validation of Menopause Quick 6 (MQ6) into the Malay language

**DOI:** 10.1186/s12875-024-02426-0

**Published:** 2024-05-18

**Authors:** Anusha Manoharan, Megat Muhammad Harris, Beh Hooi Chin, Koh Wen Ming, Zamzurina Asmuee, Norafini Salamon, Jerampang Pefer, H. Radhiyah, M. Nadia Hamimmah, Susan Goldstein, Shamala Ramasamy, Chandrashekhar T. Sreeramareddy

**Affiliations:** 1 Klinik Kesihatan Bandar Botanic, Bandar Botanic, Selangor, Malaysia; 2 Klinik Kesihatan Jinjang, Kuala Lumpur, Malaysia; 3 The Department of Primary Care, University Malaya, Kuala Lumpur, Malaysia; 4 Klinik Kesihatan Rawang, Rawang, Malaysia; 5 Klinik Kesihatan Kuang, Kuang, Malaysia; 6 Klinik Kesihatan Jalan Merbau, Sarawak, Malaysia; 7 Klinik Kesihatan Tanah Puteh, Sarawak, Malaysia; 8 Klinik Kesihatan Tanglin, Kuala Lumpur, Malaysia; 9 Klinik Kesihatan Putatan, Sabah, Malaysia; 10 Department of Family and Community Medicine, University of Toronto, Toronto, Canada; 11 Department of Psychology, International Medical University (IMU), Kuala Lumpur, Malaysia; 12 Department of Community Medicine and Public Health, International Medical University (IMU), Kuala Lumpur, Malaysia; 13 Centre for Translational Research, Institute for Research Development and Innovation, International Medical University, Kuala Lumpur, Malaysia

**Correction: BMC Prim. Care 25, 95 (2024)**

10.1186/s12875-024-02342-3

Following publication of the original article [1], the authors reported errors in the main body text. Errors were listed in the below table. The original article has been corrected.

**Table:** 

Incorrect text	Correct text
1. Abstract:	
**i. Background** Inquiring conservative Asian women about their menopausal symptoms is often challenging in crowded primary healthcare clinics. Furthermore, the subject matter is culturally sensitive to most Malaysian women. Hence, the translation of MQ6 into Malay is crucial to enable self-administration, eliminating the necessity for interviewers and mitigating potential respondent shyness.	**i. Background** Inquiring conservative Asian women about their menopausal symptoms is often challenging in crowded primary healthcare clinics. Furthermore, the subject matter is culturally sensitive to most Malaysian women. Hence, the translation of *the* MQ6 into Malay is crucial to enable self-administration, eliminating the necessity for interviewers and mitigating potential respondent shyness.
**ii. Results** The outcome of the validation was satisfactory. By the Lawshe method, the content validity ratios ranged from 0.6 to 1.0 and the content validity index was 0.914. The Internal consistency for MQ6(M) Cronbach’s alpha was 0.711 while Kuder-Richardson Reliability Coefficients KR20 was 0.676. Factor loading of all four items is above 0.70, indicating a well-defined structure. Whereas factor loading for three items fell within the range of 0.50–0.69 indicating a practically significant threshold for a new questionnaire.	**ii. Results** The outcome of the validation was satisfactory. By the Lawshe method, the content validity ratios ranged from 0.6 to 1.0 and the content validity index was 0.914. The Internal consistency for *the* MQ6(M) Cronbach’s alpha was 0.711 while Kuder-Richardson Reliability Coefficients KR20 was 0.676. Factor loading of all four items is above 0.70, indicating a well-defined structure. Whereas factor loading for three items fell within the range of 0.50–0.69 indicating a practically significant threshold for a new questionnaire.
**iii. Conclusion** MQ6 (M) has acceptable reliability and construct validity to be considered as a self-administered screening tool in primary care clinics in Malaysia.	**iii. Conclusion** The MQ6 (M) has acceptable reliability and construct validity to be considered as a self-administered screening tool in primary care clinics in Malaysia.
2. Main body text	
i. Section heading ‘**Menopause quick 6 malay, MQ6(M)**’	i. Should be ‘**Menopause** **Quick 6** **Malay, MQ6(M)**’
ii. … MQ6 was proposed as a quick and efficient tool to check for the presence of menopausal symptoms and to assist the physicians make treatment decisions based on an algorithm [12]. However the original tool has not yet been validated to date.	ii. … MQ6 was proposed as a quick and efficient tool to check for the presence of menopausal symptoms and to assist the physicians *to* make treatment decisions based on an algorithm [12]. However the original tool *had* not yet been validated.
iii. …from the author and publisher, we translated and adapted MQ6 to suit the local population’s sociocultural context….	iii. ….from the author and publisher, we translated and adapted *the* MQ6 to suit the local population’s sociocultural context….
iv. Section heading: **Translation of MQ6 English to the malay language**	iv. Should be **Translation of MQ6 English to the** **Malay language**
v. ….Women who consented completed the survey questionnaire containing demographic information, medical history, and MQ6(M)….	v. ….Women who consented completed the survey questionnaire containing demographic information, medical history, and *the* MQ6(M)….
vi. Table 2 Header: ‘**Reliability statistics of MR6 (M) items’**	**vi.** Should be ‘**Reliability statistics of** **MQ6 (M)** **items’**
vii. The reliability statistics of each of the MR6(M) items are shown in Table 2. The overall reliability of the scale by Cronbach’s alpha was 0.711 while KR20 was 0.676. Test-retest reliability was conducted within two-week interval. The established index was between 0.8 for one item and 1.0 for the other six items of MQ6(M).….	vii. The reliability statistics of each of the *MQ6*(M) items are shown in Table 2. The overall reliability of the scale by Cronbach’s alpha was 0.711 while KR20 was 0.676. Test-retest reliability was conducted within two-week interval. The established index was between 0.8 for one item and 1.0 for the other six items of *the* MQ6(M)….
viii. Exploratory Factor Analysis (EFA) of the seven items of MQ6(M) is shown in Tables 3 and 4 shows factor loadings….	viii. Exploratory Factor Analysis (EFA) of the seven items of *the* MQ6(M) is shown in Tables 3 and 4 shows factor loadings….
ix. The survey of patients attending primary care clinics was used to validate the MQ6 originally proposed for use in primary care to assist with decisions on hormonal treatment for menopausal symptoms. MQ−6 in the original English language has not yet been validated, allowing us to translate and validate a modified version of the original MQ6 into a Malay language version i.e., MQ6(M). Our validation study demonstrated acceptable psychometric properties as a quick tool applicable in primary care settings. MQ6(M) had high content validity and repeatability. Malaysian women did not express any concerns such as non-comprehensibility or ambiguity of items in MQ6(M) or acceptable reliability.	ix. The survey of patients attending primary care clinics was used to validate the MQ6 originally proposed for use in primary care to assist with decisions on hormonal treatment for menopausal symptoms. *The MQ6* in the original English language *has* not yet been validated, allowing us to translate and validate a modified version of the original MQ6 into a Malay language version i.e., MQ6(M). Our validation study demonstrated acceptable psychometric properties as a quick tool applicable in primary care settings. *The* MQ6(M) had high content validity and repeatability. Malaysian women did not express any concerns such as non-comprehensibility or ambiguity of items in MQ6(M) or acceptable reliability.
x. We should interpret the results of the validation of MR6(M) considering some limitations. First, the hospital-based study is likely not a representative sample Malaysian population in terms of ethnic distribution. Self-administered questionnaires collecting information on menopausal symptoms are subject to recall bias. Misreporting of symptoms is also possible due to emotional status, living circumstances, misconceptions about sexuality, and perceptions of ‘hot flushes’ in the tropical weather prevailing in Malaysia.	x. We should interpret the results of the validation of *the MQ6(M)* considering some limitations. First, the hospital-based study is likely not a representative sample Malaysian population in terms of ethnic distribution. Self-administered questionnaires collecting information on menopausal symptoms are subject to recall bias. Misreporting of symptoms is also possible due to emotional status, living circumstances, misconceptions about sexuality, and perceptions of ‘hot flushes’ in the tropical weather prevailing in Malaysia.
xi. In conclusion, the proposed MQ6 when translated into Malay language MQ6(M), the tool had an acceptable psychometric property such as reliability, content and construct validity. MQ6(M) with inclusion of additional item on musculoskeletal symptoms has a potential for application in primary care clinics as a quick assessment tool for menopausal symptoms among Malaysian women.	xi. In conclusion, *the MQ6(M) tool, translated into Malay language*, had an acceptable psychometric property such as reliability, content and construct validity. *The* MQ6(M) with *the* inclusion of additional item on musculoskeletal symptoms has a potential for application in primary care clinics as a quick assessment tool for menopausal symptoms among Malaysian women.
xii. **Author contributions**: Anusha Manoharan was involved in the conceptualization and design of the study, proposal preparation and ethical approval, data acquisition, and manuscript preparationMegat Muhammad Haris was involved with data acquisition and preparing the analysis dataset and manuscript writing. Beh Hooi Chin was involved with data acquisition, preparing analysis, and manuscript preparation.Koh Wen Ming was involved with data acquisition and manuscript preparation.Susan Goldstein is the original author of the MQ−6 questionnaire and was involved in the conceptualization of the project….	xii. **Author contributions**: Anusha Manoharan was involved in the conceptualization and design of the study, proposal preparation and ethical approval, data acquisition, and manuscript preparationMegat Muhammad Haris was involved with data acquisition and preparing the analysis dataset and manuscript writing. Beh Hooi Chin was involved with data acquisition, preparing analysis, and manuscript preparation.Koh Wen Ming was involved with data acquisition and manuscript preparation.Susan Goldstein is the original author of the *MQ6* questionnaire and was involved in the conceptualization of the project….
