# Supplementary material for: Translation and validation of menopause quick 6 (MQ6) into the Malay language
Source: BMC Prim Care. 2024 Mar 22;25:95. doi: 10.1186/s12875-024-02342-3 (PMC10958974; doi:10.1186/s12875-024-02342-3)
Supplement: Supplementary file 1 — Supplementary Material 1 [file 12875_2024_2342_MOESM1_ESM.docx]

Menopause Quick 6 questionnaire

Original proposed English version by Susan Goldstein available at <https://mq6.ca/mq6-assessment-tool/>

**Section E: Screening of menopausal symptoms using Menopause Quick-6 Malaysia (MQ-6M)**

Do you have?

1. Any changes in your periods? ☐ Yes ☐ No
2. Are you having any hot flushes? ☐ Yes ☐ No
3. Any vaginal dryness or pain, or sexual concerns? ☐ Yes ☐ No
4. Any bladder issues or incontinence? ☐ Yes ☐ No
5. How is your sleep? ☐ Normal sleep ☐ Disturbed sleep
6. How is your mood? ☐ Normal ☐ Changed
